# Near-Peer Teaching in Human Anatomy from a Tutors’ Perspective: An Eighteen-Year-Old Experience at the University of Bologna

**DOI:** 10.3390/ijerph19010398

**Published:** 2021-12-30

**Authors:** Ester Orsini, Marilisa Quaranta, Giulia Adalgisa Mariani, Sara Mongiorgi, Lucio Cocco, Anna Maria Billi, Lucia Manzoli, Stefano Ratti

**Affiliations:** Department of Biomedical and Neuromotor Sciences, Human Anatomy Section, University of Bologna, via Irnerio 48, 40126 Bologna, Italy; ester.orsini@unibo.it (E.O.); marilisa.quaranta3@unibo.it (M.Q.); adalgisa.mariani@unibo.it (G.A.M.); s.mongiorgi@unibo.it (S.M.); lucio.cocco@unibo.it (L.C.); annamaria.billi@unibo.it (A.M.B.); stefano.ratti@unibo.it (S.R.)

**Keywords:** near-peer teaching, human anatomy, medical school, learning, cadaver dissection

## Abstract

The University of Bologna School of Medicine in 2003 adopted a near-peer teaching (NPT) program with senior medical students teaching and assisting younger students in human anatomy laboratories. This study aimed to evaluate the effectiveness and outcomes of this program—unique on the Italian academic panorama—from the tutors’ perspective. An anonymous online survey was administered to all those who acted as peer tutors in the period from 2003 to 2021; it evaluated tutors’ perceptions regarding the influence of the tutoring experience on their skillset gains, academic performance, and professional career. Furthermore, tutors were asked to express their views on the value of cadaver dissection in medical education and professional development. The overall perception of the NPT program was overwhelmingly positive and the main reported benefits were improved long-term knowledge retention and academic performance, improved communication, team-working and time management skills, and enhanced self-confidence and motivation. Most tutors strongly believed that cadaver dissection was an invaluable learning tool in medical education, helped them to develop professionalism and human values, and positively influenced the caring of their future patients. Nearly all the participants highlighted the importance of voluntary body donation for medical education and research. The present results supported the thesis that tutors themselves benefited from the act of teaching peers; this impactful experience equipped them with a wide range of transferable skills that they could draw on as future educators and healthcare professionals.

## 1. Introduction

In the 1970s, in Italy, the number of students enrolled in the Schools of Medicine increased so much that it became impossible to organize and perform cadaver dissection activities, and, consequently, this practice was dismissed. After the national reform of the university admission system in the 1990s (law 264/1999), a limited enrollment to the degree course in Medicine and Surgery was introduced and the University of Bologna was again able to honor the Latin motto “Hic mors gaudet succurrere vitae’ (i.e., in this place death is pleased to help life). In 2003, Professor Giovanni Mazzotti informally launched a near-peer teaching (NPT) program, beginning with the class of 2001. In 2014, always thanks to his visionary thinking, a brand-new Dissecting Room was constructed and, finally, both Whole Body Donation and NPT programs had their permanent seat [1]. Nowadays, the School of Medicine at the University of Bologna offers a six-year undergraduate program that has a formal teaching of human anatomy in the second semester of the first year (Anatomy I), one in the first semester of the second year (Anatomy II), and a third one in the first semester of the fourth year (Anatomy IV). The syllabus currently includes theoretical lessons complemented by practical classes. Practical classes are aligned with each “block” of theoretical lessons and consist of surface anatomy seminars, osteology workshops, and a gross anatomy laboratory that consists of 225 hours, set-up as seven one-week sessions, attended by almost 450 students/year. These are all offered as curricular activities, held at the Institute of Human Anatomy and guided by near-peer tutors that allow for the management of such a large number of students organized into small groups. The NPT program therefore addresses the need to guarantee high-quality anatomical education in these preclinical years by means of a small group teaching format, and this is probably the reason why it is a very popular tool worldwide [2,3,4,5]. Actually, most of the recent adult learning theories drove medical school organization through significant changes. Constructivist learning theories were learner-centered and strongly supported the need for activity to promote learning. Learners were defined as “active knowledge builders” learning from and with the members of a group. Learning was not only an individual process but rather “participation is learning” [6,7,8]. According to the andragogy model (“the art and science of helping adults learn”), near-peer tutors assumed the role of “facilitator” instead of knowledge transmitter and the learners were involved in a process that included a pattern of team experiences conducted with practical methods in a comfortable environment conducive to encouraging discussion and reflection [9]. Near-peer tutors were third-to-sixth-year medical students, not professional teachers, two or more years senior to their learners. Tutors were from the same social group as tutees, helping each other to learn and learning themselves by teaching [10]. This definition emphasized how tutors’ cognitive and social congruencies with learners were key factors in the effectiveness of NPT. Such congruencies were instrumental in creating a comfortable, supporting, and encouraging educational environment for learners, who perceived a trusting relationship and a better understanding of their difficulties, and reported less fear of failure [11,12,13]. The NPT program attendance was intended as a student additional and extracurricular activity and, at the very beginning, it had just four tutors who were third-year volunteer medical students. These four students pioneered a new (at least in Italy) anatomical education tool that exponentially developed and evolved over the years, becoming a consolidated preclinical reality of the School of Medicine involving almost 400 students. The increasing number of active tutors had to be ascribed to the fact that, each year, newly recruited tutors joined the cohort, including tutors active since their third year throughout graduation year. The NPT program was coordinated by an academic group composed of professors as well as laboratory technologists and one tutor advisor. Every year, peer-tutors were carefully selected by faculty staff among those who attended a summer overseas dissection course and on the basis of various criteria, such as the score reported in the end-of-course test, the marks obtained in anatomy examinations, and motivation and enthusiasm for the field of anatomy. They were gradually introduced to tutor training programs that were longitudinal and spanned three years until graduation. Training sessions led by professors of human anatomy focused on anatomic knowledge, dissection techniques, and safety procedures for embalming and handling bodies by means of practical demonstration. Tutors developed knowledge and skills through professor shadowing and then practice, repetition, and teaching. During training sessions, professors sensitized tutors to ethical aspects of using willed bodies in medical education and instilled the fundamental value of respect for the donor’s gift. Tutoring was meant as participation and learning and it was a continuous process: these senior students were in charge of instructing freshmen students during anatomy laboratories, and, at the same time, they learned. Tutoring activities were organized into five macro-areas: surface and topographic anatomy, musculoskeletal system, thorax, abdomen and pelvis, and neuroanatomy. Tutors were allocated to a specific macro-area/group according to their attitudes, dissection skills, and expertise and were classified either as “junior” or “senior” according to the experience gained over the years, being the senior in charge of didactic coordination. In the weeks preceding gross anatomy laboratories, tutors were asked to carry out, under faculty guidance, dissections of a specific anatomic region and to draw up a textual summary of the basic content for tutees. All gross anatomy laboratories were based on such cadaveric prosections. Students, divided in small groups of 6–8 persons each, rotated to different cadaver specimens, in order to view different structures, and were allowed to handle and discuss. Tutors provided tutees with a structured interactive review, informally quizzed students on structure identification and relevant anatomical relationships, included clinical correlations, and answered to questions. The ratio tutor/tutee was of two tutors for a maximum of six to eight tutees at one time.

The literature was extremely short on evidence regarding the use of NPT programs within human anatomy education in Italian medical schools. Some Italian Universities had well-established body donation programs and had developed anatomical education and surgical training with cadavers for medical students and residents [14,15]. However, despite some reports of successful students’ NPT experience in anatomy, to our knowledge, none have discussed its use in Italy from a tutors’ point of view. The present paper aimed to profile the University of Bologna medical school NPT program by surveying the perspective of a group of students who acted (in the past and currently) as near-peer tutors within the anatomy laboratories. It focused on tutor perception of their tutoring and learning experience, the influence such experience had in their professional career, the possible effectiveness in terms of mutual and long-term benefits, and the challenge to successfully meet teaching competencies. Moreover, it sought to evaluate outcomes of the eighteen-years-long University of Bologna NPT program, to our knowledge, the only human anatomy program in Italy.

## 2. Materials and Methods

In this study, data from those who have acted as tutors over an eighteen-year period were collected, and the perceptions of their tutoring experience were analyzed, as well as the effect of tutoring on their own learning and careers.

### 2.1. Participants

The study population consisted of current medical students at the School of Medicine of the University of Bologna, residents, and medical specialists, acting or who have acted as near-peer tutors in anatomy laboratories. The group consisted of 348 tutors who were surveyed by a questionnaire.

### 2.2. Survey Design

A voluntary anonymous survey was developed and distributed to all those who tutored during the period 2003–2021, and all data obtained in this study were from this one-time survey. Invitations were sent by email to all 348 tutors in April 2021 and the email included a detailed description of the study along with a link to the questionnaire. An online questionnaire was built using Google Forms, a free online tool from Google that allows users to create forms and surveys. It was conducted in Italian and a translated version is available in Table 1. The introductory page provided a brief description of the questionnaire along with the aim of the study. The questionnaire was divided into six sections and included both closed-ended and open-ended questions. Closed-ended questions were based on a four-point Likert scale with responses ranging from very much (strong fit between self-perception and the specific item) to not at all (no fit at all). The first section included demographic information such as age, gender, and place of birth; the second section collected information regarding academic and professional background, including score gained on the admission test to medical school, graduation date, chosen medical specialty, score gained on the admission test to medical specialty school, and current job position). Questions about the tutoring experience were addressed in the third section. The fourth section included questions about the tutors’ opinion on cadaver dissection. The final section offered spaces for free observations and suggestions/comments. Participants received no compensation. The questionnaire was deactivated on 31 July 2021. The study, managing no sensitive nor personal or clinical data, obtained ethical approval by the University of Bologna School of Medicine review board. The study was conducted in agreement with EU-GDPR and the Helsinki Declaration. All data were collected anonymously (EU-GDPR, last line of whereas 26), stored, and analyzed without any possibility to identify the students. Student participation was voluntary and without any compensation (Helsinki Declaration, art.25), and they were given full explanation about the aims and contents of questionnaires (Helsinki Declaration, art.26).

### 2.3. Quantitative Analysis

Basic descriptive statistics (means, standard deviations, frequency counts, and percentages) were used to describe the sample of participants, as well as to summarize responses to quantitative questions. No formal statistical analysis was performed. For descriptive analysis, results were described as respondents disagreeing with a statement (included both “strongly disagree” and “disagree” responses) and agreeing with a statement (included both “agree” and “strongly agree” responses).

### 2.4. Qualitative Analysis

Responses to open-ended questions were analyzed using the qualitative data analysis software NVivo to assess word frequency. Text responses were translated to English and imported into NVivo; word clouds were created to visually represent the most frequently used words. Font sizes were proportional to word frequency (i.e., more common words are presented with larger fonts, and fewer common words are presented with smaller fonts). All narrative comments were grouped for themes (based on surveys’ main sections) and then each theme was categorized into three sub-themes (emerging from analysis of open-ended responses and focusing on one notable salient element). Each subtheme was supported by a representative example comment.

## 3. Results

### 3.1. Participation

Three hundred and forty-eight tutors served as NP tutors between 2003 and 2021 in the anatomy course at the School of Medicine of the University of Bologna. Considering the entire period, a total of 287 replies were received with a return rate of 82.5%. It was assumed that participants who did not respond were mostly tutors from past academic years who had already completed their medical education at the time of data collection and whose contact details had in the meantime changed.

### 3.2. Evaluation of Quantitative Data and Open-Ended Queries

Based on the topics the survey questions dealt with, it was possible to group them and to identify three main themes: (I) impact on education, academic achievement, and career (queries 1 to 7 and open-ended question A); (II) improvement of professional and personal skills (queries 8 to 11 and open-ended question B); (III) impact of cadaver dissection on learning and professional development (queries 12 and 13 and open-ended question C). Survey queries and percentages of responses are summarized in Table 1.

#### 3.2.1. Impact on Education, Academic Achievement, and Career

**Figure 1:**
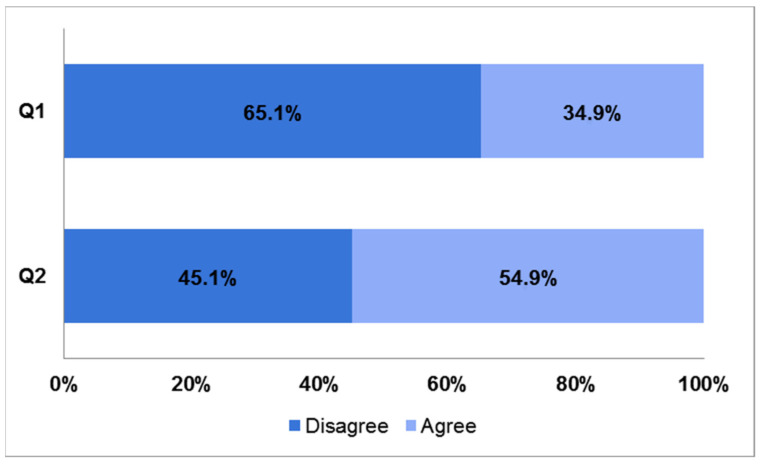
Bar charts showing responses to questions 1 and 2 of the survey. Q1: I had a clear career path plan when I entered medical school. Q2: My career path plan has been affected by near-peer tutoring experience.

The survey asked tutors to reflect on their intended career plans at the beginning of their medical education path, and 65.1% had a vague or no idea about their careers (Q1), but a slight majority of them (54.9%) also felt that their career plan has been affected by near-peer tutoring experience (Q2) (Figure 1).

**Figure 2:**
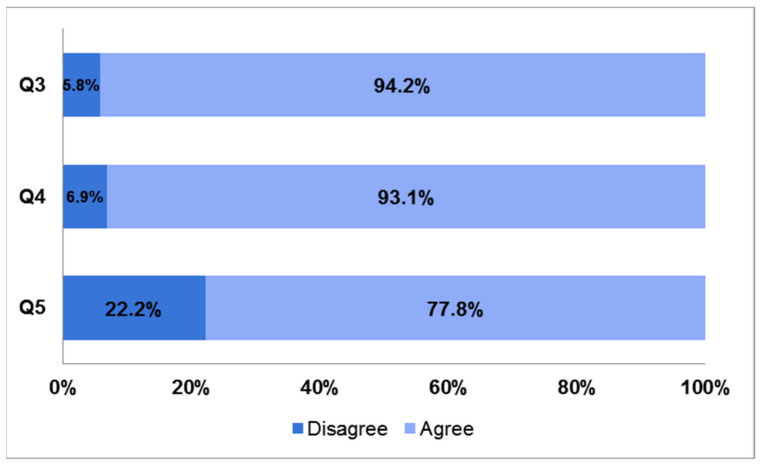
Bar charts showing responses to questions 3, 4, and 5 of the survey. Q3: Attending/graduating at University of Bologna medical school has brought an added value to my education. Q4: In the case of positive response to the previous question, it was my experience as a near-peer tutor that mainly affected it. Q5: I noticed differences in attitudes and preparation among colleagues who were not enrolled as near-peer tutors.

The majority (94.2%) agreed that attending/graduating at the University of Bologna medical school brought an added value to their education (Q3) and a similar percentage (93.1%) believed that this was affected by their near-peer tutoring experience (Q4). More than 70% of respondents (77.8%) pointed out differences regarding preparation and academic performance with colleagues who did not have the opportunity to tutor (Q5) (Figure 2).

Tutors were asked to retrospectively rate their appreciation with the NPT model prior to being enrolled as tutors, i.e., as tutees, (Q6), and nearly 100% felt satisfied or very satisfied with this model in human anatomy teaching (98.6%), as shown in Table 1. When asked if they would recommend near-peer tutoring experience to early-years medical students (Q7), the overwhelming majority of peer tutors (99.3%) either agreed or strongly agreed with the query (see Table 1).

**Figure 3:**
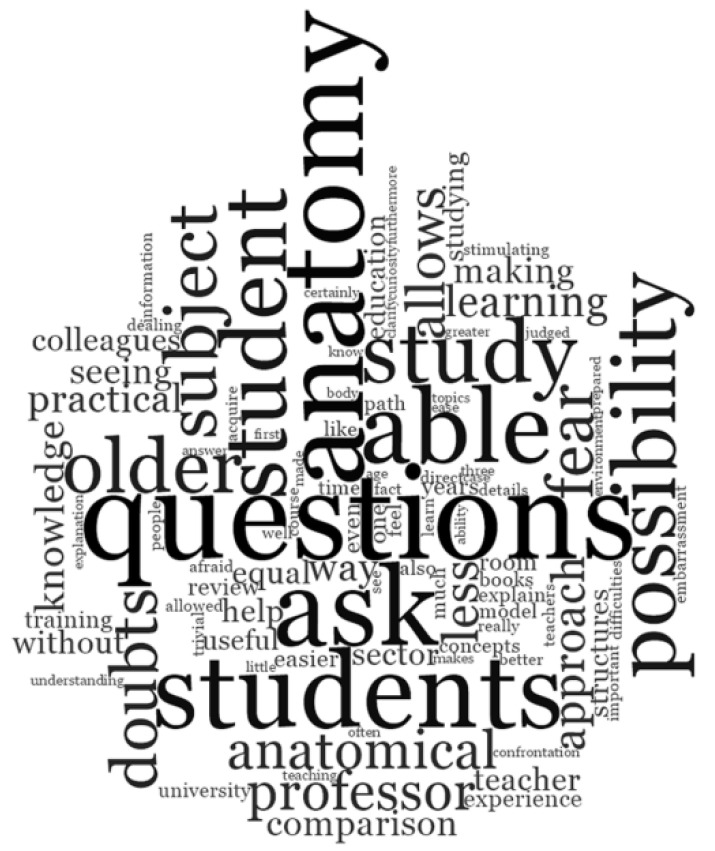
Word cloud representing tutors’ responses to the open-ended question: “What were the strengths of this near-peer tutoring model?”

In response to the open-ended question “What were the strengths of this near-peer tutoring model?” the words commonly mentioned were “questions,” “able,” “ask,” “possibility,” “students,” “fear,” and “doubt,” as shown by the larger text (Figure 3).

More specifically, all the participants showed general enthusiasm to this open-ended question. Most of them responded that there were many strengths related to this method of teaching anatomy, specifically in relation to the possibility of asking questions freely and without fears, of interacting with professors and colleagues to solve doubts, and of increasing their own anatomical knowledge and practical skills (Table 2).

#### 3.2.2. Improvement of Professional and Personal Skills

**Figure 4:**
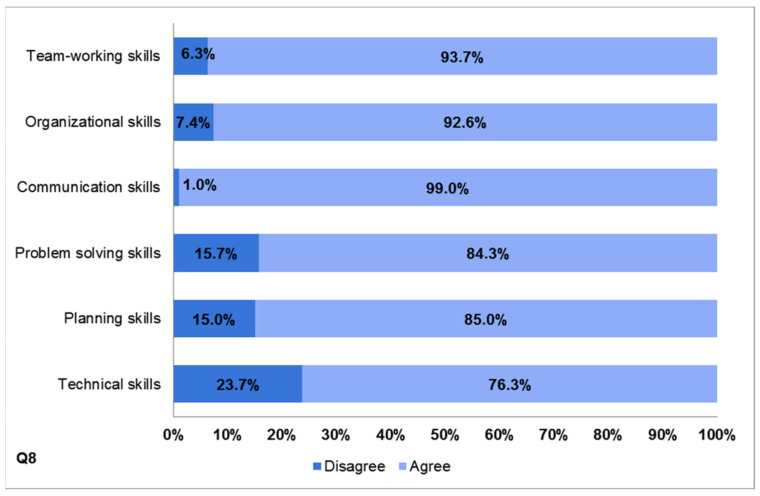
Bar chart displaying results of self-assessment in skills development (Q8).

The authors sought to identify the effect of peer tutoring on the development of a broad range of specific competencies. Tutors were asked to recognize skills acquired by participating in the NPT program (Q8). They were able to select multiple skill choices. Overall, there was a high level of positive responses to all items of the category “development of skills.” More in detail, 93.7% reported that they acquired team-working skills, 92.6% organizational skills, 84.3% problem-solving skills, and 85% planning skills. Interestingly, nearly all tutors (99%) agreed or strongly agreed that the tutoring experience promoted the development of communication skills. A higher percentage of disagreement (23.7%) was found in relation to the development of technical skills (Figure 4).

Questions 9, 10, and 11 dealt with additional long-term outcomes that could be reaped within the peer teaching sessions. Of note, an overwhelming majority of respondents reported that they had gained other positive benefits from the tutoring experience; as shown in Table 1, 93% said that the opportunity to constantly revise anatomy allowed them to consolidate their own knowledge and to improve their exam performance (Q9); 91% recorded increased self-confidence (Q10) and 90.2% felt that this experience has helped them to develop and maintain a proactive approach to academic achievements (Q11).

**Figure 5:**
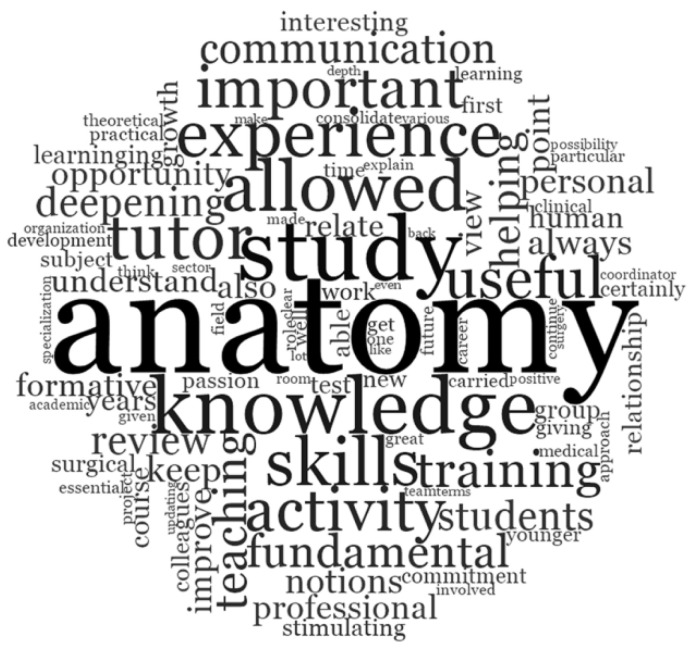
Word cloud representing tutors’ responses to the open-ended question: “Describe the role near-peer tutoring experience plays/played in your professional career”.

Figure 5 shows a word cloud generated from the tutors’ responses to the free text question” Describe the role near-peer tutoring experience played in your professional career,” and the words that appeared more frequently were “knowledge,” “skills,” “training,” “communication,” “opportunity,” and “useful.”

More specifically, all the participants also showed strong interest to this open-ended question. Most of them described that their near-peer tutoring experience allowed them to develop many relevant skills useful in their professional career (Table 2).

#### 3.2.3. Impact of Cadaver Dissection on Learning and Professional Development

**Figure 6:**
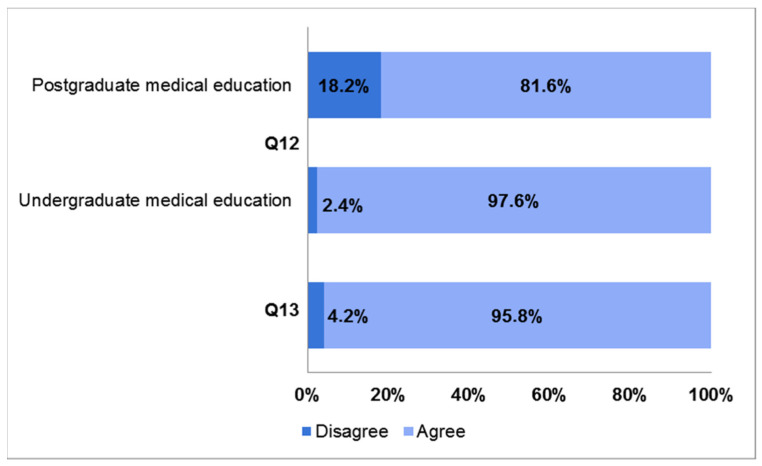
Bar charts showing responses to questions 12 and 13 of the survey. Q12: “Cadaver dissection is an invaluable and necessary learning tool in undergraduate/postgraduate medical education”. Q13: “Cadaver dissection is a powerful tool in positively shaping professional and ethical values like compassion, empathy and respect toward patients”.

The last section of the survey collected tutors’ opinions on the importance of cadaver dissection in medical education and their perceptions toward the body donation program. Regarding Q12, most of the participants stated that cadaver dissection had an important or very important value in undergraduate medical education (97.6%). Slightly divergent from the surveys of the students, a lower percentage considered it important or very important in postgraduate medical education (81.6%), with the percentage of disagreement or strong disagreement being 18.2% (Figure 6).

**Figure A1:**
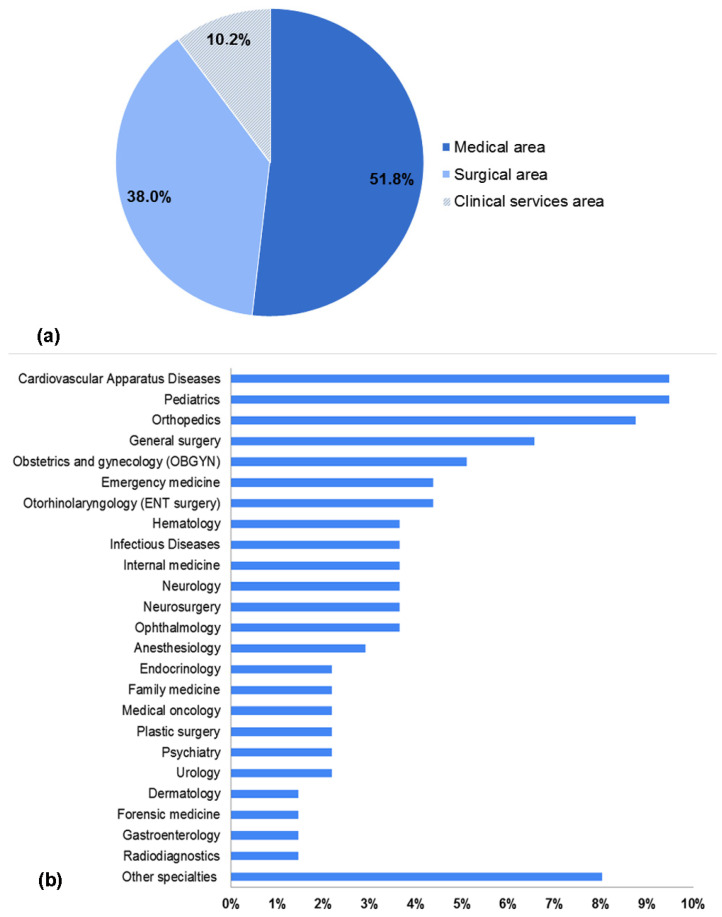
Medical specialties chosen by past and current tutors. (**a**) Pie chart showing selected medical specialties by macro-areas; (**b**) top medical specialties chosen by tutors. Other specialties chosen by the 8% of participants included anatomical pathology, audiology and phoniatrics, food science and human nutrition, maxillofacial surgery, microbiology and virology, nephrology, occupational medicine, pediatric surgery, physical medicine and rehabilitation, and respiratory apparatus diseases.

Among those who disagreed, residents/specialists in the medical area represented the majority (59.4%), followed by those in the surgical area (9.4%) and in the clinical service area (6.2%); the remaining 18% of statements were from fresh graduates; thus, their feedback was considered not assessable (data not shown). Tutors were also asked to indicate their medical specialty choice, and the query has been raised considering that, in Italy, medical specialty schools are grouped into three broad areas: medical, surgical, and clinical services (see Table A1 in Appendix A). Regarding this medical specialty choice, 51.8% were in medical area specialties, 38.0% in surgical area specialties, and 10.2% in clinical services area specialties (see Figure A1a in Appendix A). The most frequently selected specialties were cardiovascular apparatus diseases and pediatrics (9.5%), orthopedics (8.8%), and general surgery (6.6%), followed by OBGYN (5.1%) and emergency medicine (4.4%) (see Figure A1b in Appendix A). Finally, in Q13, an overwhelming majority (95.8%) of respondents stated that cadaver dissection had helped them to develop professionalism, as well as a humanistic approach to patient care (Figure 6).

**Figure 7:**
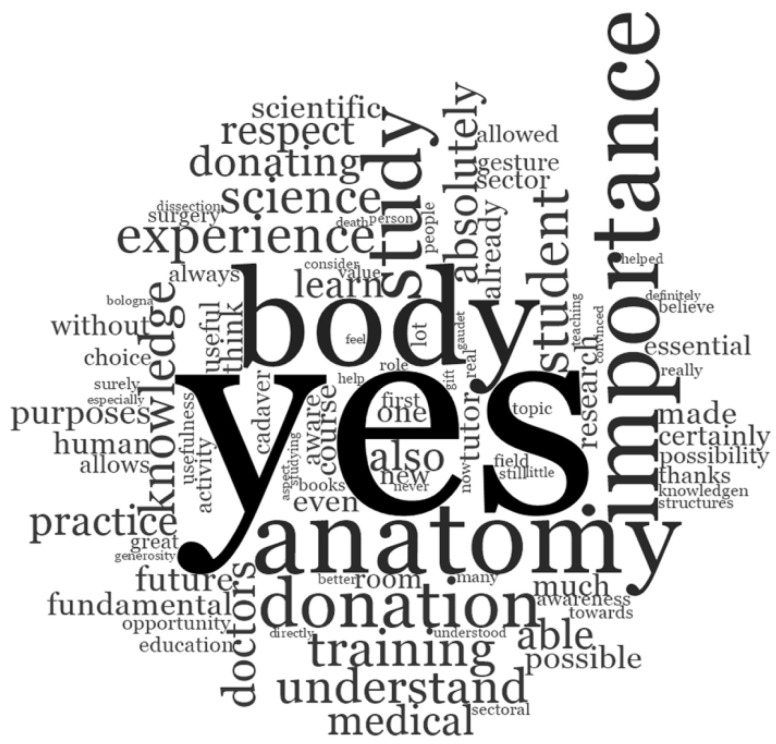
Word cloud of tutors’ responses to the open-ended question: “Do you feel that near-peer tutoring experience has/had positively influenced your perception towards body donation for medical education and scientific purposes?”.

In response to the narrative question” Do you feel that near-peer tutoring experience has/had positively influenced your perception towards body donation for medical education and scientific purposes?” the words commonly mentioned were “yes,” “body,” “knowledge,” “experience,” and “importance” (Figure 7).

More specifically, all the participants answered positively to this open-ended question. Most of them described that their near-peer anatomy tutoring experience was not only useful for their medical education and medical career, but also for understanding the importance of body donation (Table 2).

## 4. Discussion

The present study was designed to profile the University of Bologna medical school NPT program using a web-based survey that targeted the medical students who acted as near-peer tutors within the anatomy laboratories over an eighteen-year period (2003–2021). Such a model proved to be a reasonable education method allowing to overcome the relatively low faculty-to-student ratio and improving interactions between participants [16,17,18,19]. The eighteen-year duration of the program created the opportunity to explore its features in terms of tutors’ perspective and perception of their tutoring and learning experience, the influence such an experience had in their professional career, the possible effectiveness in terms of mutual and long-term benefits, and the challenge to successfully meet teaching competencies. Remarkably, the high level of motivation shown by peer-tutors and the deep emotional connection built with faculty staff throughout the undergraduate years facilitated the information collection, even from some of those tutors who had already graduated at the time of survey administration, and, therefore, the return rate was 82.5%.

### 4.1. Impact on Education, Academic Achievement, and Career

Respondents in this study reported that they value NPT as an educational strategy that impacts on their education, academic achievement, and career. As their near-peer activity was not part of the formal curriculum and was completely voluntary, it could be assumed that this experience was driven by intrinsic motivation: the possibility to understand and, therefore, to express preferences regarding the area of activity with respect to their individual attitudes and strengths; the opportunity to share their clinical experience with younger students who were starting their medical preclinical training; the chance to enjoy in a very comfortable atmosphere a unique experience, both educationally and socially [20]. A large majority of tutors recognized the University of Bologna NPT program as an added value and acknowledged that such near-peer teaching experiences provided them with academic benefits, derived from gaining deeper knowledge and developing new skills. They perceived themselves as better prepared than colleagues not involved in the NPT project. Nearly all respondents retrospectively appreciated, as tutee, the model of near-peer teaching in the preclinically oriented anatomy course and, as tutor, would recommend the experience to students. Even open-ended question A (What were the strengths of this near-peer tutoring model?) feedback demonstrates that the students undertaking the role of tutor at the University of Bologna medical school found the program to be a useful tool in effectively teaching human anatomy in small groups. Some of the most mentioned words were “questions,” “able,” “ask,” “possibility,” “students,” “fear,” and “doubt”. These words emphasized that respondents, who performed the dual role of near-peer tutee and, later on, of anatomy tutors, recognized and valued the cognitive and social congruence of peer tutors as effective key factors present in the undergraduate medical NPT program. The present findings were consistent with current theories already reported in the literature [12,21,22,23,24] according to which near-peer education is effective due to cognitive and social congruence. Such congruence was instrumental in creating a comfortable, supporting, and encouraging educational environment for learners who perceived a trusting relationship and a better understanding of their difficulties, and reported less fear of failure [11,13].

### 4.2. Improvement of Professional and Personal Skills

A similar trend of results was observed on NPT as an effective educational tool strategy, which improves professional and personal skills. There was overwhelming support for peer teaching in helping to develop a broad range of specific competencies. Regarding the perceived skillset gains, the achievement of communication skills had the largest percentages of respondents either agreeing or strongly agreeing (99%), followed by team-working (93.7%) and organizational skills (92.6%), with these three being the more teaching-related skills. These results matched with the positive evidence authors have witnessed over the years and showed that, by means of the NPT model, tutors acquired transferrable personal and professional skills. During the preparatory session led by professors of human anatomy, tutors gained experience by means of practical demonstration, practice, and repetition. Over time, they became more effective at communicating, gained insight into the educational needs of learners, and were able to develop and organize course content. Through professor shadowing, they learned about techniques and resources that could be applied to ensure efficient team-working. Interestingly, the authors’ experience indicated that the more motivated the tutors were, the more personal resources such as time, energy, patience, and enthusiasm they put into tutoring activity. These data were particularly interesting in that the NPT program did not explicitly teach these skills, but it was the anatomy tutor experience in its entirety to be strongly perceived as formative. The finding was important because the development of communication skills is essential not only for physicians who choose an academic career and therefore will play a teaching role, but for all physicians providing patient care [25,26,27,28]. Being able to effectively communicate with patients about illnesses and treatment is crucial and is a challenging skill that can be acquired and improved through practice in near-peer teaching. Similar findings were already described in several previous studies investigating peer tutoring in anatomy [2,26,29,30,31,32,33,34]. The necessity, as core competencies, of these teaching-related skills has been reaffirmed by many international regulatory bodies. Even the World Health Organization (WHO) strongly recommended that a central aim of medical education should be strategies imparting effective communication skills among students [35], which was actually one of the benefits reported by this study. A lower percentage of agreement resulted for technical skill development (76.3%). Certainly, this was a recurrent theme within student feedback: they often complained about the limited time for practicing dissection and the lack of opportunity to actively dissect. Authors were already aware of this drawback. It may be ascribed to the insufficient number of cadavers and the lack of awareness about the usefulness of body donation in Italy, but, in that regard, the recent law no.10/20 published under the title “Regulations about the disposition of post mortem human body and tissues for study, training and scientific research purposes” and committed to the mission of “education” will be helpful in promoting communication campaigns. Moreover, in September 2021, the Institute of Human Anatomy of the University of Bologna was officially designated by the Ministry of Health as a reference center for the management of deceased body preservation and use. Other very valuable perceived outcomes were related to the opportunity to constantly revise anatomy, to increase self-confidence, and to develop a proactive approach to academic achievements. Authors strongly believed NPT was an educational strategy in which near-peer tutors were responsible for teaching younger students, and this triggered an active learning process. The benefits were mutual and manifold, as experienced by tutors: the activity was itself a powerful driver of learning as it not only involved knowledge organization and consolidation but forced one to broaden access to personal skills (time management, leadership, self-confidence, self-consciousness, and working collaboratively) according to a process that, in turn, resulted in reinforcement and better long-term retention of anatomical knowledge [36,37,38,39,40,41,42]. Open-ended question B (Describe the role near-peer tutoring experience played in your professional career) responses were consistent with the above discussed data. Some of the most mentioned words were “knowledge,” “skill,” “training,” “communication,” “opportunity,” and “useful”. These replies indicated that tutors perceived NPT as a very rich learning experience: the act of tutoring other students had a positive impact on themselves too and improved self-confidence and skills that they could apply throughout their careers [38,43,44,45,46].

### 4.3. Impact of Cadaver Dissection on Learning and Professional Development

Other questions posed in this study were specific to the impact of cadaver dissection on learning and professional development. The great majority of the participants stated that cadaver dissection had a positive impact on the development of humanistic values such as empathy and compassion and contributed significantly to their professional formation. These results showed that the use of cadavers was useful not only for teaching anatomy but also in instilling in medical students the ethic and humanistic values that define the core nature of their future medical practice. Cadaver dissection caused medical students to reflect on matters such as illness, death, and a humanistic approach to their future dying patients; furthermore, contact with death for the first time gave them the opportunity to learn how to handle emotions, to increase their motivation to cure and to care, and to build a positive patient–doctor relationship [47,48,49,50].

Even tutors’ opinions pertaining to the invaluable importance of cadaver dissection in undergraduate medical education were strongly positive. However, 18.2% of the respondents disagreed or strongly disagreed with its role on postgraduate medical education. The majority of disagreeing participants were residents/physicians in nonsurgical specialties, which inevitably assigned different importance to anatomical knowledge and hands-on training on cadavers compared to surgical specialists [51]. The cadaver-based anatomy lab played a key role in preclinical medical educational curricula and was crucial for teaching undergraduate medical students. It allowed them to gain basic knowledge and competences, to engage in tactile learning, to appreciate the consistency and the texture of human tissues, and to observe pathological conditions, anatomical abnormalities and variations while promoting pre-surgical training [1].

Responses to the narrative question “Did near-peer tutoring experience positively influence your perception towards body donation for medical education and scientific purposes?” suggested that the program fulfilled its aim of creating a “culture” of body donation. The words commonly mentioned were “yes,” “body,” “knowledge,” “experience,” and “importance,” and, notably, more than fifty percent of the sentences started with the word “Yes.” The word “knowledge” was repeatedly encountered but with different meanings. In addition to its combination with “anatomical,” “human body,” and “subject,” it was also used to mean “be aware of”: many participants reported that they had little or no knowledge about body donation in Italy before their tutoring experience. All tutors expressed deep appreciation and gratitude to body donors, and they felt honored and privileged to have learned anatomy from these “silent teachers” [52,53]. This feedback demonstrated that the goal of transmitting empathy and respect for body donors was achieved.

### 4.4. Limitations and Future Research

The impact of the NPT experience on medical specialty choice was not clearly established, as the expected direct causality with surgical specialties or other anatomically related fields did not apparently occur. However, this result did not necessarily and directly reflect a lesser interest in gross and surgical anatomy among respondents who selected nonsurgical specialties. It was well known that specialty selection was a complex, multifactorial process and young doctors selected a medical specialty not only according to their own attitudes, aspirations, and vocational interests but even based on the availability of residency positions (in Italy, about one in four available positions was in the surgical area). The data presented here reflected a wide variety of specialties chosen by students who acted as tutors, but the authors did not have a “control population” (a cohort of medicine students that did not act as tutors) nor information about the total number of residency positions in each specialty (medical, surgical, and clinical services) in Italy/year, and this was a limitation of the present study. Another limitation was the extreme heterogeneous cohort: respondents were at different stages in their medical careers at the time of the survey. Some respondents were medical students recently recruited as tutors, while others were senior tutors about to graduate, residents, or specialists who completed the NPT experience several years ago. It was conceivable that the current position of the respondents might have influenced their answers to the survey. Therefore, further investigation will be required to clarify these issues, to properly interpret NPT program effectiveness and efficiency, and to better understand the role that it should play in medical education. Formal statistical analysis also still needs to be performed, and the volume of demographic data and its influence on tutors’ perceptions need to be explored (see Appendix B).

## 5. Conclusions

Near-peer education programs are nowadays commonly established worldwide. Nevertheless, the literature was extremely short on evidence regarding the use of NPT programs within human anatomy education in Italian medical schools. To our knowledge, the University of Bologna program is unique on the Italian academic panorama. As shown by the present study that reported the perceived benefits of NPT from the perspective of the tutors, this eighteen-year-old program has grown with time, becoming a form of a supplemental educational approach in the early preclinical years aiming to improve the quality of learning both for tutees and tutors. It was strongly based on social and cognitive congruence and provided an interesting opportunity to work in small groups, thus establishing a student-centered education. It allowed one to further develop anatomical knowledge, to acquire comprehensive competencies that resulted in key skills for future careers, and to positively shape fundamental ethical values of future physicians. Finally, human anatomy near-peer teaching not only helped tutors to develop educational, professional, and personal skills, but also helped them to raise awareness regarding the theme of body donation, still little widespread in Italy. Data from this research offered grounds for continually evaluating students’ perception and experiences in order to address their needs and to successfully implement such a strategic educational resource.

## Figures and Tables

**Table 1:** Summary of survey questions and responses.

Questions and Statements		SD	D	A	SA
**Impact on Education, Academic Achievement and Career**					
Q1	I had a clear career path plan when I entered medical school	26.1%	39.0%	24.1%	10.8%
Q2	My career path plan has been affected by near-peer tutoring experience	12.7%	32.4%	37.7%	17.2%
Q3	Attending/graduating at University of Bologna medical school has brought an added value to my education	0.7%	5.1%	35.2%	59.0%
Q4	In the case of positive response to the previous question, it was my experience as a neer-peer tutor that mainly affected it	0.4%	6.5%	45.6%	47.5%
Q5	I noticed differences in attitudes and preparation among colleagues who were not enrolled as near-peer tutors	3.0%	19.2%	47.7%	30.1%
Q6	As a tutee, prior to being enrolled as tutor, I appreciated/enjoyed the near-peer teaching model in human anatomy education	0.7%	0.7%	15.2%	83.4%
Q7	I would recommend near-peer tutoring experience as a beneficial tool in preclinical education pathway to early years medical students	0.0%	0.7%	12.3%	87.0%
A	What were the strengths of this neer-peer tutoring model?	Open-ended question			
**Improvement of professional and personal skills**					
Q8	Near-peer tutoring experience helped me to develop the following skills: ▪ Team-working skills ▪ Organizational skills ▪ Communication skills ▪ Problem solving skills ▪ Planning skills ▪ Technical skills				
		1.1%	5.2%	36.2%	57.5%
		0.7%	6.7%	41.8%	50.8%
		0.0%	1.0%	14.3%	84.7%
		1.7%	14.0%	44.2%	40.1%
		2.1%	12.9%	47.4%	37.6%
		3.2%	20.5%	30.0%	46.3%
Q9	The opportunity to constantly revise anatomy helped me to improve my academic performance	0.7%	6.3%	43.9%	49.1%
Q10	Near-peer tutoring experience helped me to increase my self-confidence	0.7%	8.4%	41.5%	49.4%
Q11	Near-peer tutoring experience helped me to develop a proactive approach to the degree course	0.0%	9.8%	37.1%	53.1%
B	Describe the role near-peer tutoring experience plays/played in your professional career.	Open-ended question			
**Impact of cadaver dissection on learning and professional development**					
Q12	Cadaver dissection is an invaluable and necessary learning tool in: ▪ undergraduate medical education ▪ postgraduate medical education				
		0.0%	2.4%	20.8%	76.8%
		2.2%	16.0%	35.4%	46.2%
Q13	Cadaver dissection is a powerful tool in positively shaping professional and ethical values like compassion, empathy and respect toward patients	0.6%	3.6%	30.9%	64.9%
C	Did near-peer tutoring experience positively influence your perception towards body donation for medical education and scientific purposes?	Open-ended question			

Abbreviations: SD, strongly disagree; D, disagree; A, agree; SA, strongly agree.

**Table 2:** Summary of the narrative comments grouped into themes and subthemes.

Themes	Subthemes	Example Comments
Impact on education, academic achievement, and career	Education	The hands-on experience has allowed a better memorization of what has been studied in books.
	Academic achievement	Seeing colleagues only a few years older able to master such a complex subject in such a clear and confident way helped me to deal with it in a different way when preparing for the exam and contributed to the considerable interest in the subject that I still study today.
	Career	I consider my experience as anatomy tutor as a fundamental part of my professional career. I have not only been able to follow several students during their second year of studies, but I have also been able to have a concrete example of interdisciplinary collaboration and functionality, fundamental in my work.
Improvement of professional and personal skills	Practical skills	It prepares for what the hospital reality is and promotes the development of clinical thinking and technical skills. It makes you extremely prepared and allows you to interact during internships.
	Attitude	It allowed to understand the importance of anatomical-clinical correlations and to develop teaching and communication skills; the organization of teaching activities contributed to the development of organizational skills.
	Knowledge	It has certainly consolidated my knowledge, giving me an overview of the topographical anatomy otherwise difficult to acquire with the theoretical study only, which helps me a lot in my current clinical work, especially in the interpretation of diagnostic images. It also allowed me to get in touch with the world of research and conferences at an early stage.
Impact of cadaver dissection on learning and professional development	Awareness of body donation	Before my experience as a student in the dissecting room, I did not even know that there was the possibility of studying anatomy on cadaver in Italy.
	Effectiveness of body donation	Yes. Studying anatomy on cadaver and not only in atlases is essential to fully and deeply understand it. Donating one’s body for such purposes is a way to promote the education of future doctors and therefore the quality of public health.
	Ethical value of body donation	Yes. Body donation is a gesture of great generosity and unconditional trust in medical science which contributes to the advancement of medical education and biomedical research. Treating donors and our future patients with the highest level of respect, care and dedication will be our concrete forms of gratefulness.

## Data Availability

All data generated or analyzed during this study are included in this published article.

## Appendix A

In Italy, medical specialty schools are grouped into three broad areas: medical, surgical, and clinical services. Each area includes different classes, and these latter gather into several types, as shown in the following table.

Specialty schools last four to five years; to practice family/general medicine, doctors must complete a three-year postgraduate training course in family medicine accessible through public exam.

**Table A1:** Medical specialties divided into areas, classes, and types.

MEDICAL AREA	SURGICAL AREA	CLINICAL SERVICES AREA
Class of GENERAL AND SPECIALISTIC CLINICAL MEDICINE Internal medicine Emergency medicine Geriatrics Sports medicine Thermal medicine Medical oncology Community medicine and primary care Allergy and clinical immunology Dermatology Hematology Endocrinology Food science and human nutrition Digestive system diseases Cardiovascular apparatus diseases Respiratory apparatus diseases Infectious diseases Nephrology Rheumatology Class of NEUROSCIENCES AND CLINICAL BEHAVIOURAL SCIENCES Neurology Child neuropsychiatry Psychiatry Class of CLINICAL MEDICINE OF DEVELOPMENTAL AGE Pediatrics	Class of GENERAL AND SPECIALIST SURGERY General surgery Pediatric surgery Plastic surgery Obstetrics and gynecology (OBGYN) Orthopedics Urology Class of HEAD AND NECK DISTRICT SURGERY Maxillofacial surgery Neurosurgery Ophthalmology Otorhinolaryngology (ENT surgery) Class of CARDIOTHORACIC AND VASCULAR SURGERY Cardiac surgery Thoracic Surgery Vascular surgery	Class of DIAGNOSTIC AND LABORATORY MEDICINE Anatomical pathology Microbiology and virology Clinical Pathology and Biochemistry Class of DIAGNOSTIC IMAGING AND RADIOTHERAPY Radiodiagnostics Radiotherapy Nuclear medicine Class of SPECIALIST CLINICAL SERVICES Anesthesiology, critical care, and pain medicine Audiology and phoniatrics Physical medicine and rehabilitation Class of BIOMEDICAL SPECIALIST CLINICAL SERVICES Medical genetics Pharmacology and clinical toxicology Class of PUBLIC HEALTH Hygiene and preventive medicine Occupational medicine Forensic medicine Medical Statistics and Biometry Class of DENTISTRY SPECIALTIES Oral surgery Orthodontics Pediatric dentistry Class of PHARMACY Hospital pharmacy Class of HEALTH PHYSICS Medical physics

The following graphs show the areas of specialization (a) and the type of specialty chosen by tutors enrolled in the study (b).

## Appendix B. Participants’ Demographics

Among the 287 tutors who completed the questionnaire, 151 (52.6%) were females and 136 (47.4%) were males. The mean age of the participants was 26.2 ± 3.8. A small number (2.4%) were non-Italian, while the majority (97.6%) were Italian; of these, 69.7% were from Northern Italy, 13.2% from Central, and 17.1% from Southern and insular Italy. One hundred nineteen respondents (41.5%) were medical students and 168 (58.5%) were postgraduates. The majority of medical students (89.9%) attended the degree course in Medicine and Surgery in Italian, while 10.1% attended the international course of Medicine and Surgery, entirely taught in English. Most of postgraduates were residents (106; 63.1%) and 18.45% (31) were medical specialists. The remaining participants were fresh graduates who did not indicate their choice, because, at the time of data collection, they were preparing to or had just run the national admission test to specialty residency and were waiting for the rankings to come out.
